# Patterns of Heart Rate Dynamics in Healthy Aging Population: Insights from Machine Learning Methods

**DOI:** 10.3390/e21121206

**Published:** 2019-12-09

**Authors:** Danuta Makowiec, Joanna Wdowczyk

**Affiliations:** 1 Institute of Theoretical Physics and Astrophysics, University of Gdansk, Wita Stwosza 57, 80-308 Gdańsk, Poland; 2 First Department of Cardiology, Medical University of Gdansk, Debinki 7, 80-211 Gdańsk, Poland; joanna.wdowczyk@gumed.edu.pl

**Keywords:** heart rate variability, entropy, fragmentation, aging in human population, factor analysis, support vector machines classification

## Abstract

Costa et. al (Frontiers in Physiology (2017) 8255) proved that abnormal features of heart rate variability (HRV) can be discerned by the presence of particular patterns in a signal of time intervals between subsequent heart contractions, called RR intervals. In the following, the statistics of these patterns, quantified using entropic tools, are explored in order to uncover the specifics of the dynamics of heart contraction based on RR intervals. The 33 measures of HRV (standard and new ones) were estimated from four hour nocturnal recordings obtained from 181 healthy people of different ages and analyzed with the machine learning methods. The validation of the methods was based on the results obtained from shuffled data. The exploratory factor analysis provided five factors driving the HRV. We hypothesize that these factors could be related to the commonly assumed physiological sources of HRV: (i) activity of the vagal nervous system; (ii) dynamical balance in the autonomic nervous system; (iii) sympathetic activity; (iv) homeostatic stability; and (v) humoral effects. In particular, the indices describing patterns: their total volume, as well as their distribution, showed important aspects of the organization of the ANS control: the presence or absence of a strong correlation between the patterns’ indices, which distinguished the original rhythms of people from their shuffled representatives. Supposing that the dynamic organization of RR intervals is age dependent, classification with the support vector machines was performed. The classification results proved to be strongly dependent on the parameters of the methods used, therefore determining that the age group was not obvious.

## 1. Introduction

The cardiac tissue of the human heart is under the constant influence of the autonomic nervous system (ANS), the part of the nervous system that works largely without our consciousness. There are two branches of ANS, the sympathetic and vagal subsystems, which acting oppositely, the sympathetic increasing and vagal reducing the heart rate, control the homeostasis in the cardiovascular system, i.e., the proper supply of nutrients to each cell of the organism [1,2]. The maintenance of a stable heart rhythm involves different reflex feedback mechanisms, which makes the whole phenomenon complex. With age or with disease, a gradual impairment of the functioning of the complex interplay between these mechanisms could develop [3,4,5].

There are methods like measurement of norepinephrine spillover, microneurography, and imaging of cardiac sympathetic nerve terminals that can give information about the actual state of ANS [4]. However, it turns out that changes in the activity of ANS reveal themselves in the time intervals between heartbeats, in the dynamics of so-called RR intervals [6,7]. Partially, this is due to the fact that the activities of sympathetic and vagal subsystems differ in response delay [2,8]. The effect of the vagal system can be seen immediately in the same heart beat or in the next beat in case of a tetanic stimulation [9]. The response of the sympathetic activity is assumed to occur within a few seconds and lasts for a few seconds [8]. This way, the analysis of heart rate fluctuations, called heart rate variability (HRV), has become a noninvasive technique, which potentially can be used to assess ANS activity.

The ANS control over the heart is strong in the sense that it dominates all other possible sources of heart rhythm variation, including tissue remodeling, especially at the initial stage [5]. The tissue remodeling due to inflammation or fibrosis could lead to abnormal rhythms, called also erratic rhythms [10], which with time could develop into arrhythmia.

Many efforts have been made in the aim of getting through HRV as much information as possible on the functioning of ANS and the state of cardiac tissue [5,11,12,13,14,15]. Standard studies use the global indices of variability such as the standard deviation of RR intervals or the power of specific oscillations in the RR interval signal. In particular, it has been found that the presence or absence of some oscillations, called low frequency, is associated with sympathetic activity, while others, called high frequency, with vagal activity. However, the relation between the variations in RR intervals and the control mechanisms or other aspects possibly influencing the heart rate is still not explained. After more than thirty years of this research, disappointment has developed; see [16] for the critical review. The criticism refers to their weak repeatability and/or weak predictability. Furthermore, a vivid discussion is taking place on the meaning of HRV [17,18]. Thus, HRV, its physiological background, and diagnostic benefits still require careful elucidation and wait for verification of both the concept and methods of estimating.

The dynamics of changes in RR intervals can be represented symbolically as a sequence of accelerations and decelerations [19,20]. It has turned out that short term patterns, constructed as short subsequences of the sequence of accelerations and decelerations, could be a good source for studying the relationship between events that shape the HRV. Especially, their relation to the vagal tone has been established [20]. Recently, Costa et. al [21] proposed to symbolize the RR intervals by patterns that were supposed to discern the abnormality of heart rhythm related with the emergence of erratic rhythms. Consequently, the concept of fragmentation and fragmentation measures has been developed.

In the following, we investigate the characterization of RR intervals provided by the fragmentation measures (indices relying on counting specific events), especially by comparing their performance to the corresponding entropy measures (indices built to quantify the distribution of the counted events). Together, we show results obtained from other standard HRV indices, known to describe the short term variability. In total, 33 HRV measures were used to describe the HRV during the nocturnal rest of 181 healthy subjects of different ages from twenty years old to octogenarians. We assumed that the signals of healthy people at different ages should provide the ability to extract the specificity of heart rate dynamics with healthy aging.

An enormous progress in machine learning achievements, together with their excellent implementations on user-friendly platforms [22], pushed many of us to test whether this new methodology can help in explaining the phenomenon of HRV and in the diagnosis of cardiovascular diseases [23,24]. Traditional machine learning (ML) is close to the statistical methods of data analysis where each item in the dataset, here a four hour signal, is described by a set of features [25]. However, it is also said that “machine learning is statistics minus any checking of models and assumptions” [26]. This is because implementations of many ML algorithms can be effective even when the data are gathered without a carefully controlled experimental design and in the presence of complicated nonlinear interactions. Because of that, sometimes, ML is located as the common domain between hackers and traditional mathematical statistics [27].

HRV of a given subject can be expressed using several measures. Many of them are related either by mathematical formulas or by the concept of the physiological phenomenon they describe. Which one to choose for analysis? ML techniques allow considering all of the measures, called ML features, and investigating the relationships between them. In the following, we practice with two ML techniques: exploratory data analysis and classification. In particular, we applied the exploratory factor analysis to identify possible hidden variables driving a given set of features. The classification task was performed with support vector machine (SVM). SVM is a supervised learning model that has a clear theoretical background, which is important in the case of reading the results.

Moreover, we also benefit directly from the ML flexibility. As the validation for the obtained results, we propose to consider outcomes arising from the analysis of surrogate signals. The surrogates were provided by random shuffling of the real RR interval signals. Random shuffling destroys the time patterns; however, it preserves the distribution of RR intervals. We assumed that this way, we could filter out the patterns of the specific dynamics that was present only in the the real signals, from the overall statistical relations.

The article is organized as follows. We start with the presentation of the study group of subjects, the methods of ECG recording, and the construction of RR interval signals in Section 2.1. The description of the HRV indices together with the relationship between fragmentation measures and corresponding entropic measure are presented in Section 2.2 and Section 2.3. Section 2.4 is for the propagation of ML methods in the HRV analysis. An introduction to exploratory factor analysis and to classification with SVM is provided. In Section 2.5, the specification of the statistical methods used is given. The results and their discussion are presented in Section 3. Subsequently, in Section 3.1, the outcomes of the factor analysis together with their interpretations are given. In Section 3.2, we show and discuss observations obtained from investigations of entropic measures. Finally, we test whether SVM methods are able to display changes emerging with biological aging better than the classical regression methods. These results are given in Section 3.3. Section 4 contains the summarizing discussion and closing remarks.

## 2. Methods

### 2.1. Data Acquisition

Healthy volunteers meeting the following inclusion criteria [28]: age 18–89 years old and sinus heart rhythm in ECG, were included in the study. The exclusion criteria were as follows: presence of ischemic heart disease, heart failure, hemodynamically significant valvular heart disease, multi-drug controlled hypertension, or the presence of abnormalities in additional tests indicating organ complications of hypertension, the presence of symptomatic atherosclerosis or its features in physical examination, a history of atrial fibrillation or other arrhythmia during Holter recording, significant disorders of atrioventricular and intraventricular conduction in ECG, diabetes and other diseases significantly affecting the phenomenon of sinus rhythm variability, taking medications that significantly affect the sinus node, the presence of numerous artifacts in the 24 h electrocardiographic Holter recordings, nicotinism of more than 5 cigarettes a day, pregnancy, and finally, no consent to participate in the study. Prior to the enrollment, in order to confirm sinus rhythm and exclude abnormalities indicating cardiovascular diseases, a 12 lead electrocardiogram was recorded. Volunteers were then subjected to echocardiographic examination, which evaluated the occurrence of possible organ complications of hypertension, as well as other abnormalities implying the presence of cardiovascular diseases. In the next stage, twenty four hour recording of the electrocardiographic signal was carried out using the Digicorder 483 digital recorders from Delmar and Lifecard from Delmar Reynolds. The study was approved by the Ethic Committee of the Medical University of Gdansk (NO. NKEBN/142-653/2019).

The recordings were analyzed on the Delmar Reynolds system (SpaceLabs Healthcare, USA). The sampling rate of ECG was 128 Hz, which ensured 8 ms accuracy for the identification of R-peaks in the QRS complex. The quality of the ECG recordings and accuracy of R-peak detection were verified by visual inspection by experienced cardiologists. All normal beats were carefully annotated, so that only normal sinus rhythms were considered in our investigations.

In total, 181 signals were analyzed. The set of recordings was divided into groups corresponding to the age decade of a person: 20’s (30 subjects: 17 women), 30’s (21 subjects: 11 women), 40’s (33 subjects: 13 women), 50’s (31 subjects: 13 women), 60’s (27 subjects: 12 women), 70’s (22 subjects: 10 women), 80’s (17 subjects: 11 women).

The period of nocturnal rest was discerned individually, in each recording separately, according to the appearance of consecutive hours with a low heart rate. From each recording, the four hour signal with normal-to-normal RR intervals {RR(n):n=0,⋯,N} was extracted. All gaps were annotated, which was used in the construction of a series of patterns, namely only consecutive in time RRintervals were mapped to a signal of RR actions {δRR(n)=RR(n)−RR(n−1):n=1,⋯,N}. Small gaps of a size of one or two missing values were filled with medians from the surrounding {−3,+3} neighbors. The extra editing procedure was applied to RR actions as follows: if the difference δRR between two consecutive RR intervals was larger than 300 ms or smaller than −300 ms, then this δRR was replaced by the interval 300 ms, −300 ms, respectively.

### 2.2. Entropic Measures of HRV

For each signal with RR intervals {RR(n)} and its signal of RR actions {δRR(n)}, the series of decelerations, accelerations, or no action is defined as follows:

(1) $$\left\{\delta RR\left(n\right)=\left\{\begin{array}{ccc} >0, & \mathrm{deceleration} & :d\\ <0, & \mathrm{acceleration} & :a\\ =0, & \mathrm{no}\;\mathrm{action} & :0 \end{array}\right.,\;n=1,\cdots ,N\right\}$$

The fragmentation indices of Costa et al. [21] were designed to collect the information about the presence of specific short segments of accelerations and/or decelerations, which were supposed to show the essence of heart rate dynamics. In particular, the probability of segments of two alternating actions: ad and da or three alternating actions: ada and dad was of interest. Similarly, the short sequences with the same actions: aaa or ddd were found important in the description of heart rate dynamics. The following definitions were applied by us:

- Percent of inflection points: PIP=[p(ad)+p(da)]100%
- Percent of alternation segments: PAS=[p(ada)+p(dad)]100%
- Percent of short segments: PSS=[1−p(aaa)−p(ddd)]100%

It was obvious that the symbolization (1) depended on the resolution of a signal. Moreover, this symbolization did not take into account the size of an action, whether the action was strong or weak. Because each resolution of a recording provides natural quantization to the recorded values, let us use the resolution Δ of a given signal of RR intervals to represent the space $\Pi_{1}$ of its quantified RR actions:

(2) $$\delta RR\left(n\right)\in \left\{-M\Delta ,\cdots ,-\Delta ,\;0,\;\Delta ,\cdots ,M\Delta \right\}\;\mathrm{where}\;M=\max_{n}\left\{\frac{\left|\delta RR(n)\right|}{\Delta }\right\}$$

Accordingly, the spaces of two or three subsequent in time actions can be considered:

$$\begin{array}{ccc} \Pi_{2} & = & \{(\delta RR(n),\delta RR(n+1))\}=\{(i,j):|i|,|j|\leq M\},\\ \Pi_{3} & = & \{(\delta RR(n),\delta RR(n+1),\delta RR(n+2)))\}=\{(i,j,k):|i|,|j|,|k|\leq M\}, \end{array}$$

with constant *M* defined as in (2). The three spaces $\Pi_{1}$, $\Pi_{2}$, and $\Pi_{3}$ were finite and for each signal different. They collected the quantified patterns of the short term dynamics of the heart beats of a given person. The probabilistic structure of these spaces can be estimated by the Shannon entropy,

$$\begin{array}{ccc} E_{1} & = & -\sum_{i\in \Pi_{1}}p\left(i\right)\ln p\left(i\right)\\ E_{2} & = & -\sum_{\left(i,j\right)\in \Pi_{2}}p\left(i,j\right)\ln p\left(i,j\right)\\ E_{3} & = & -\sum_{\left(i,j,k\right)\in \Pi_{3}}p\left(i,j,k\right)\ln p\left(i,j,k\right) \end{array}$$

It is easy to see that if the RR actions occur independently of each other, then $E_{2}=2E_{1}$ and $E_{3}=3E_{1}$, while $E_{1}$ attains its maximal value.

The stochastic features of the short term dynamics can be evaluated by [29]:

- entropy of transition rates $\;S_{T}=E_{1}-E_{2}$
- self-transfer entropy $\;sTE=\left(E_{2}-E_{3}\right)-S_{T}$

The entropy of transition rates $S_{T}$ evaluates a given system dynamics as if it were a Markov chain [30], i.e., memoryless dynamics driven by a table of transition rates. It has been proven that $S_{T}$ is equal to approximate entropy [31], a popular nonlinear metrics used in HRV, however applied to RR intervals. If elements of the analyzed signal are independent of each other, then $S_{T}=E_{1}$. The self-transfer entropy sTE, the notion based on transfer entropy [32], accounts for the influence of the past on the current action. It estimates memory effects that are not encoded in a transition matrix of a Markov chain model. In case of a signal with independent elements sTE=0.

The fragmentation measures are based on counting events ignoring the distribution of events. Thanks to the entropic approach, the relevance of particular fragmentation patterns can be included. Accordingly, let us consider indices based on the partial entropy, i.e., on the entropy related to the distribution of the particular patterns of accelerations and decelerations:

$$\begin{array}{ccc} E_{\left(ad\right)} & = & -\sum_{-i,j=1,\cdots ,M}p\left(i,j\right)\ln p\left(i,j\right)\\ E_{\left(da\right)} & = & -\sum_{i,-j=1,\cdots ,M}p\left(i,j\right)\ln p\left(i,j\right)\\ E_{\left(ada\right)} & = & -\sum_{-i,j,-k=1,\cdots ,M}p\left(i,j,k\right)\ln p\left(i,j,k\right)\\ E_{\left(dad\right)} & = & -\sum_{i,-j,k=1,\cdots ,M}p\left(i,j,k\right)\ln p\left(i,j,k\right)\\ E_{\left(aaa\right)} & = & -\sum_{-i,-j,-k=1,\cdots ,M}p\left(i,j,k\right)\ln p\left(i,j,k\right)\\ E_{\left(ddd\right)} & = & -\sum_{i,j,k=1,\cdots ,M}p\left(i,j,k\right)\ln p\left(i,j,k\right) \end{array}$$

The fragmentation indexes ignore also the presence of non-action events. We will observe the role of these events, counting their appearance as n${}_{\mathrm{zero}}$.

### 2.3. The Set of Considered HRV Measures

The standard HRV measures are usually grouped according to the methods of their computations: time domain, frequency domain, or nonlinear measures; see [11,15] for the definitions and interpretation. Furthermore, they are often divided due to the supposed phenomena they describe: short term correlations or long term correlations [16].

Here, the following standard time domain measures were considered: the average of all RR intervals (meanRR), the average of all heart rates (meanHR), standard deviation of all RR intervals (stdRR), square root of the mean of the sum of squares differences between adjacent RR intervals (RMSSD), the percentage of differences between adjacent RR intervals that are longer than 50 ms (pNN50) and longer than 20 ms (pNN20). The frequency domain HRV measures relied on estimation of the power spectral density computed with the Lomb–Scargle periodogram. The frequency bands were: for very low frequency (VLF, 0.003–0.04 Hz), low frequency (LF, 0.04–0.15 Hz) and high frequency (HF, 0.15–0.4 Hz). The frequency domain measures were extracted from the power spectral density for each frequency band and relative powers of VLF (rVLF), LF (rLF), and HF (rHF). Additionally, the two nonlinear measures arising from the Poincare plot: sd1 and sd2, were included also; see [33] for the definition.

In total, thirty three HRV measures were included in a set of features used in the ML analysis. Many of them are known to be correlated, as for example meanRR and meanHR. Nevertheless, we considered them to see how mathematical relationships translate into the correlation analysis. For further discussion, we grouped the HRV indices according to the known properties they describe or the mathematics involved:

- general: meanRR, meanHR
- long term dependence: stdRR, sd2
- short term dependence: pNN50, pNN20, RMSSD, sd1
- frequency: total, rVLF, rLF, rHF
- fragmentation: PIP, PAS, PSS
- partial fragmentation: p(ad), p(da), p(ada), p(dad), p(aaa), p(ddd)
- dynamic landscape: $E_{3}$, $E_{2}$, $E_{1}$, $S_{T}$, sTE
- partial entropy: $E_{ad}$, $E_{da}$, $E_{ada}$, $E_{dad}$, $E_{aaa}$, $E_{ddd}$
- no action counts: n${}_{\mathrm{zero}}$

The vector of 33 features $\left\{f^{\left(i\right)}=\left(f_{\mathrm{meanRR}}^{\left(i\right)},\cdots ,f_{n_{\mathrm{zero}}}^{\left(i\right)}\right)\right\}$ was estimated for each of 181 signals. We studied features of the full recording, of 240 min. Furthermore, the same features were calculated for segments of the recording, here 5 min segments, though any other segmentation was possible. A set of all 5 min segments of one person, namely 48 items, was taken into account. Moreover, we considered statistics found for physiologically justified extremes of the segmented 5 min features. In particular, the segments representing the minimum of heart rate, which could be attributed to deep sleep [12,13,34], were considered. Furthermore, the segments with the minimum of stdRR were investigated. The reduced HRV is often attributed to the transition from deep sleep to the REM phase of sleep [12].

Finally, the same analysis was performed for shuffled signals. The shuffling of RR intervals was performed ten times with the procedure random.shuffle of the numpy library of Python. Shuffling RR intervals preserved the distribution of RR intervals, but it destroyed the patterns of RR actions specific for a given system dynamics. The resulting distribution of RR actions was different because in the case of shuffled RR intervals, for any action δ, we have:

$$p\left(\delta \right)=\sum_{\left(RR,RR-\delta \right)}p\left(RR,RR-\delta \right)\;\mathrm{where}p\left(RR,RR-\delta \right)=p\left(RR\right)p\left(RR-\delta \right)$$

which leads to the maximally random distribution of RR actions for a given distribution of RR intervals.

### 2.4. Machine Learning Methods

Factor analysis (FA) and classification with support vector machine (SVM) are among the standard methods of ML based on the features [22,27]. FA is used to identify relationships among features of interest. These relationships arise based on the assumption that our observations are due to the linear relation between several hidden factors and some added Gaussian noise. Consequently, these factors can be found as the eigenvectors of the correlation matrix of features. Each vector describes the underlying relationships between the feature and the hidden factor. In the following, we considered only those factors for which the eigenvalue was greater than 1 (the Kaiser–Guttman rule).

Classification is a central goal of many ML procedures. Among the most popular feature based methods are linear discriminant analysis, random forests, gradient boosting, and SVM. All of them belong to the class of supervised learning, i.e., methods that build the classification by learning the data. SVM has a clear intuitive interpretation, at least in the linear case. The SVM method constructs a classification decision function by optimization of the margin, i.e., the area at the decision function. The points that are closest to the decision boundary are called the support vectors. Therefore, it has a clear intuitive interpretation in the case when the decision function is linear. In the following, we limited our investigations to SVM.

SVM can be used with kernels to solve the nonlinear classification. The most popular kernels are Gaussians, which estimate the distance between any pair of feature points $f^{\left(i\right)},f^{\left(j\right)}$ as $k\left(f^{\left(i\right)},f^{\left(j\right)}\right)=\exp\{-\gamma ||f^{\left(i\right)}-f^{\left(j\right)}{\left|\right|}^{2}\}$. Accordingly, they are tuned by the value of parameter γ: the cut-off for the Gaussian ball. Depending on γ, the classification can be quite general (large γ) or more specific for the studied signals (small γ). In the following, we assumed γ=0.2, which is smaller than γ=0.5 of the default procedure setting. “C” is the second regularization parameter of the SVM kernel procedures. It trades between the correct classification and maximization of the decision function’s margin. Our estimates used C = 1, which is a default value of the applied numerical methods. With the above settings, we obtained the stable classification results. Eventually, by the SVM, we were given the posterior probability for each data point to belong to a given class [35]. These probabilities will be presented as the mean ± std of 50 runs.

All estimates were done with homemade Python scripts. We used the Python libraries: factor_analyzer packet [36] and from scikit learn [37]: sklearn.svm.SVC for numerical estimates and matplotlib for visualization of the results.

### 2.5. Statistical Methods

For each feature separately, the linear regression by least squares: $index=a_{0}+a_{1}age$, was estimated in order to detect their dependence on age. The quality of the regression was evaluated by $R^{2}$ and the *p*-value of the estimated coefficients. Within that test, the analysis of variance (Holm–Sidak method for pairwise comparison), the normality test (Shapiro–Wilk), and the equal variance test (Brown–Forsythe) were performed. In case the normality test failed, the Kruskal–Wallis one way analysis of variance on ranks was performed with Dunn’s method applied for pairwise comparison.

The SigmaPlot 13.0 software (Systat Software, Inc., San Jose, CA, USA) was utilized in all tests. The results were confronted with estimates provided by generalized least squares (Python libraries [38], namely: statmodels.api.GLS, statmodels.stats.anova, statsmodels.formula.api.ols).

## 3. Results and Their Possible Interpretation

### 3.1. Factor Analysis of 240 min Recordings

The FA was performed on the set of features when the values of each feature were normalized. The FA identified five groups: the hidden factors, which could be supposed to drive the set of observed features. The relationships among the features and factors found in the 240 min signals are presented in Table 1. Each of the considered features depended on each factor. However, the strength of this dependence significantly changed from one factor to another factor. For each HRV index, in bold, we point at the factor that drove the given index, namely the feature related to the factor with the biggest value. For comparison, the factor analysis results obtained for shuffled signals are displayed in parentheses.

It turns out from Table 1 that the maximal values for the considered measures were greater than 0.65, often close to one, indicating the crucial role played by the given factor on the given index. Moreover, these values were distinct from the values obtained for shuffled signals. Following this idea, we grouped the most important features for each factor. In the case of physiological signals, these groups can be interpreted as follows:

- The last column of Table 1, the column of Factor V, is concentrated at indices: stdRR and sd2, assumed to measure the long term correlations.
- The previous column, Factor IV with domination of meanRR and meanHR, corresponds to the so-called general stability measures. The personal specificity of the cardiac tissue cells can be thought as driving these indices.
- The third factor is cumulated on specific fragmentation indices; PSS, p(ddd), p(aaa), corresponding to the partial entropic measure $E_{ddd}$, $E_{aaa}$, and the low frequency spectrum rLF. Sequences of increases or decreases in a heart rate are commonly related to the activity of the sympathetic branch of ANS. Furthermore, rLF is assumed as a standard index of the sympathetic activity. Therefore, this factor can be referred to as the index of sympathetic activity.
- Factor II refers to the two fragmentation indices: PAS and PIP, and related to them, the partial fragmentation indices. Furthermore, all corresponding partial entropy measures were strongly related. Because of the concentration on the alternation patterns, this factor can be seen as revealing the mechanisms of maintaining the balance in ANS control.
- Finally, the first factor can be interpreted as driving the short term dependence. It influences the standard measures of short term correlations (pNN50, pNN20, RMSSD, sd1), all dynamical landscape measures ($E_{3}$, $E_{2}$, $E_{1}$, $S_{T}$, sTE), and rHF. Furthermore, the two action partial entropy indices: $E_{ad}$ and $E_{da}$, were driven by this factor. These findings agree with our belief that all of them display the short term correlations, which in turn might be related to the activity of the parasympathetic part of ANS. Notice that the no action counter n${}_{\mathrm{zero}}$ gained here its maximal influence, which located the index among measures of short term relations. However, the presence of the total power and rVLF must be admitted, which are rather attributed to the general power of a system (total) or long term oscillations (rVLF).

**Figure 1:**
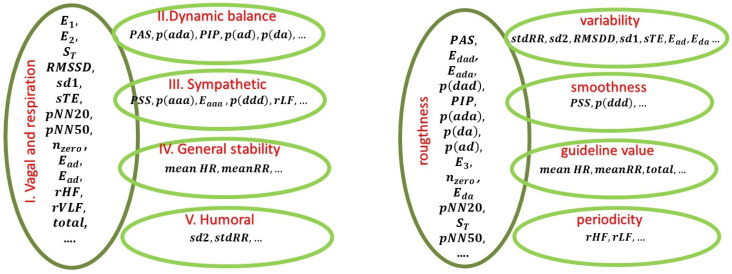
The graph of the hidden factors’ generators of the studied features identified by factor analysis (FA) in RR intervals (240 min) (**left part**) and in shuffled signals (**right part**).

The above observations can lead us to the hypothesis about the possible physiological interpretation of the five factors of FA that drive the observations in our data as follows (see Figure 1):

Factor I: vagal nervous system activity including respiration;
Factor II: mechanisms of maintaining the dynamical balance in ANS;
Factor III: sympathetic nervous system activity;
Factor IV: mechanisms responsible for the overall system stability;
Factor V: long term regulatory mechanisms that mainly are based on humoral activity.

As the dynamic landscape measures and fragmentation indices were found to belong to different factors, Factor I versus Factors II and III, respectively, then one can suggest that they represent different aspects of HRV phenomena. However, the measures concentrated on patterns with inflection points: $E_{ad}$, $E_{da}$, p(ad), and p(da) seemed to be driven by two factors: I and II. It is interesting that Factor I influenced more strongly the partial entropies: $E_{ad}$ and $E_{da}$ than the corresponding counters: p(ad) and p(da), whereas in the case of Factor II, we saw the opposite relation. Therefore, this observation might suggest that the distribution of the inflection patterns reflected rather the vagal activity, while the number of these events referred to maintaining the balance in ANS.

It turned out that the main factors governing the characteristics of shuffled signals were different from those found in the original series; see the values in parentheses in Table 1. However, again, the dominant features in each factor could be grouped and then named. This time, however, the names followed the statistical phenomena that these features represented; see Figure 1, right.

In the case when FA is limited to the set of entropic measures, i.e., indices from the dynamical landscape and from partial entropy served as the features set, then we obtained only two significant factors. The first factor contained all dynamical landscape measures and $E_{ad}$ and $E_{da}$, while the second one was concentrated on the three event partial entropies. Hence, the entropic indices were divided into the measures of the vagal activity and the remaining ones.

FA for 5 min segments (all 48 segments from each person were taken into account) provided six important factors. The long term dependencies (Factor V in 240 min signals) were moved to the first factor with short term dependencies, which could be expected because long term and short term indices now worked in similar time scales. However, it was surprising that Factor I of 240 min signals was divided into the three new factors. These new factors were the domination of the total, rVLF, and rHF (the first factor), of $E_{3}$ and $S_{T}$ (the second factor). All remaining indices of 240 min Factor I formed the third factor. One might think that the physiological and statistical components were mixed.

### 3.2. Visualization of the Stochastic Relations between Features

**Figure 2:**
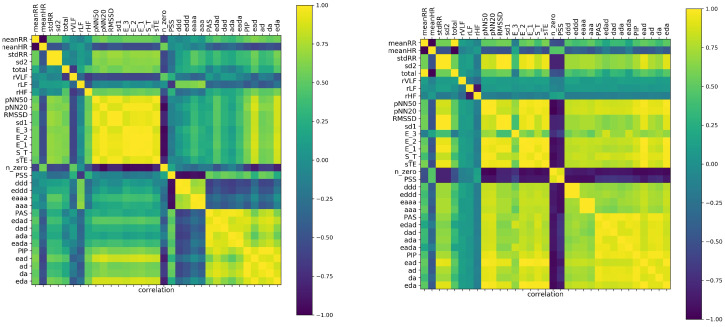
Tables of correlation values between studied features estimated from the analysis of original 240 min signals (**left**) and when the 240 min signals were randomly shuffled (**right**). One can identify factors, clusters of strongly correlated features, and then observe the correlations between different factors.

In general, if variables are strongly correlated, then we can use the value of one variable to predict the value of the other variable. This way, the correlation coefficient became a measure of dependence (at least in the statistical sense) between the features. Consequently, the correlation coefficients can be used in clustering the features. The correlation matrix on the basis of which the factors of Table 1 were identified is shown in Figure 2. As the validation for the observed correlations, we display the correlation matrix obtained from shuffled signals.

The cluster structure of the analyzed features was easily discerned. By the naked eye, in Figure 2, one can identify the factors discussed in the previous subsection. Starting from the the obvious anticorrelation between meanRR and meanHR, it is noticeable that the presence of the monotonic patterns ddd or aaa was rather anticorrelated with the appearance of the alternate patterns, ad, da, dad, and ada, and very weakly correlated with the values of the total entropy, $E_{3}$, $E_{2}$, and $E_{1}$, and dynamic measures $S_{T}$ and sTE. One can observe also how these relations changed when correlations among the features were estimated from the shuffled signals.

It turned out that in the case of shuffled signals, the time and nonlinear indices, except the general features of Factor IV, became strongly correlated. Furthermore the features estimated by Fourier analysis displayed independence from all other measures. These facts could support the hypothesis that in the case of shuffled signals, the correlation matrix revealed only the mathematical relations between features. Consequently, the comparison between correlations detected in our original signals and correlations found in the shuffled signals suggested the hypothesis that the dynamics of decelerations and accelerations were not random, but followed special patterns. This observations strongly motivated our interest in the short term patterns.

**Figure 3:**
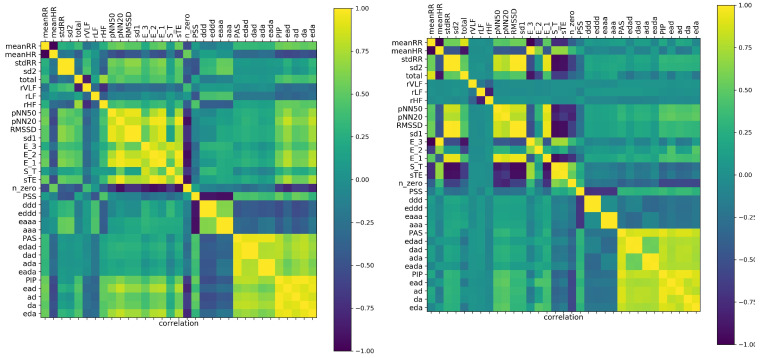
Tables of correlation coefficients between studied features estimated from the sets of real five minute signals (**left**) and when the five minute analysis is done on signals randomly shuffled (**right**). One can notice the correlations inside the factors.

In Figure 3 are displayed correlations found for five minute signals. Here, in the estimates of features for each person, we included all 48 segments of a 240 min long recording. It means that we studied correlations in a set of 33 features and with 48 × 181 = 8688 patients. Accordingly, such patients were not independent, as was demanded by statistical analysis rules. However, remaining in the spirit of ML, we accepted this violation. One can think that such analysis is like the stroboscopic observation of a system. The deep learning methods are perfectly suitable for this kind of analysis. However, this approach we leave for our future investigations.

It turned out that the set of our 33 features, observed in a stroboscopic way, provided similar factorization of measures to that one obtained in the case of estimates with the whole 240 min signals, though the values of the correlation coefficients were lower. Distinctly from the 240 min analysis, the frequency measures occurred as being independent of all others. Moreover, the large cluster consisting of short term and dynamic landscape measures revealed some intrinsic structure: the indices $E_{3}$ and $S_{T}$ were detected as independent of all other indices of the cluster.

The absence of known mathematical relations between features, as well as the appearance of surprising correlations in the shuffled signals suggested that correlation analysis could be misled by the poor information obtained from the five minute segments of signals. The local fluctuations could break the probabilistic relations in the sense that we could not see the expected dependence among the variables. An accidental variation that was actually recorded in a signal drove the estimates. Concluding, HRV outcomes obtained from five minute segments were found misleading. This problem will be investigated further in the next subsection.

### 3.3. Graphs of Strong Correlations within Entropic Measures

The entropic measures considered by us, i.e., measures that are based on total or partial entropy, are strongly mathematically related. One should expect that these relations are revealed by the correlation coefficients. If we assume that by strong correlations, we mean the correlation coefficient greater than 0.8, then the following picture of the strongly correlated features emerges from our data.

**Figure 4:**
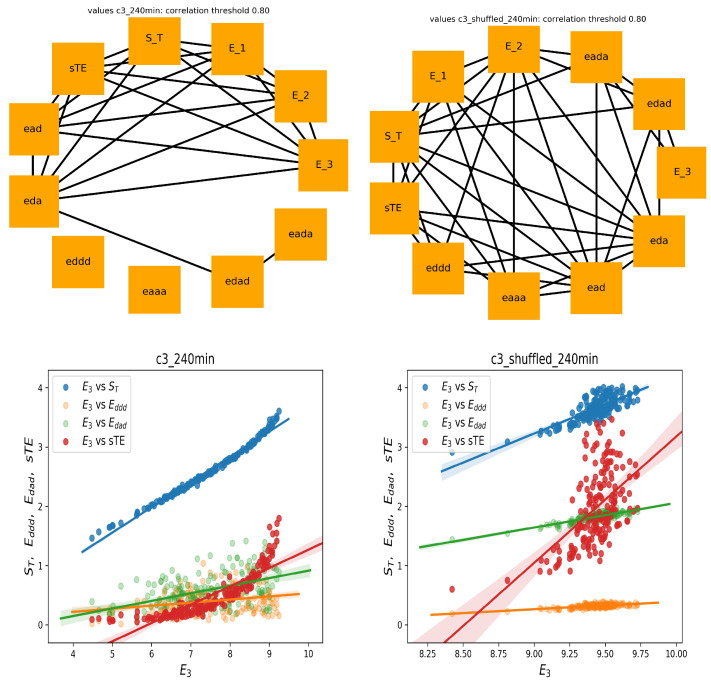
The graphs of strong correlations (θ=0.8) between entropic measures estimated from 240 min signals: original signals (**left**) and shuffled signals (**right**). Below, the scatter plots (with regression lines) between $E_{3}$ and important other entropic indices are shown.

In Figure 4, two graphs of strong correlations are plotted: for features estimated from the 240 min original signals and from the shuffled signals. Together, we show the scatter plots between $E_{3}$ and the most important for the system dynamics indices, namely of stochastic dynamics $S_{T}$ and sTE and partial entropies that construct $E_{3}$: $E_{ddd}$ and $E_{dad}$.

Evidently, all expected mathematical relations are displayed in the graph, which shows the relations obtained from the shuffled signals. This graph is almost complete: the features are strongly correlated. However, the original signals seemed to not follow the statistics. Especially, let us point at the links between $E_{3}$ and sTE and between $E_{3}$ and $S_{T}$. The strong correlations between $E_{3}$ versus $S_{T}$ and $E_{3}$ versus sTE were present only among original signals, whereas they were absent in the shuffled signals. A different relation was observed for correlations between $E_{3}$ and $E_{ddd}$ and $E_{dad}$. There was a noticeable distinction between the values of indices obtained from original signals and from shuffled signals. Additionally, the variability among these values influenced the correlation. Therefore, one can see the structure of the correlations obtained from the original signals as specific for the dynamics of the studied physiological system.

**Figure 5:**
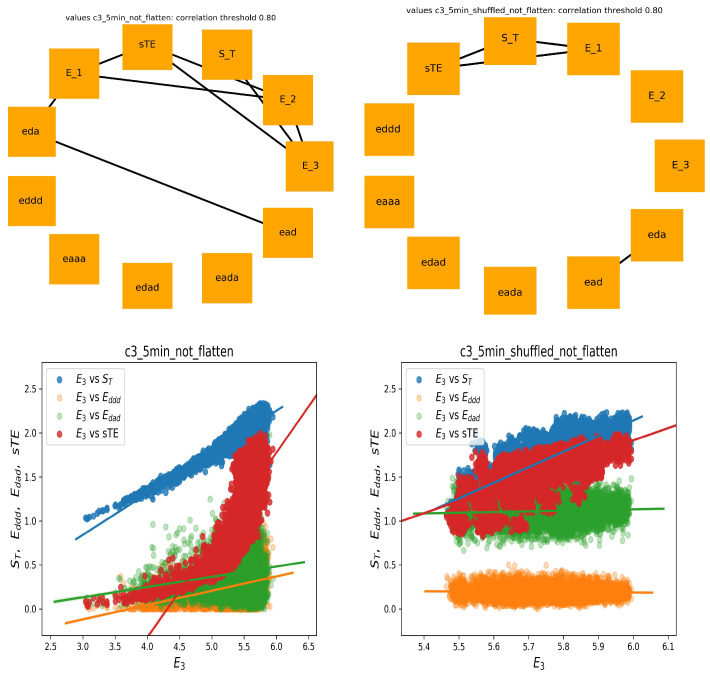
The graphs of strong correlations (θ=0.8) between entropic measures estimated from all five minute segments of studied signals: original signals (**left**) and shuffled signals (**right**). Below, the scatter plots (with regression lines) between $E_{3}$ and other important entropic indices are shown.

On the other hand (see Figure 5), in the case of shuffled signals divided into five minute segments, and when each feature was represented by 48 values, we obtained an almost empty graph of strong correlations. Notice the difference in the dispersions of values of the displayed features in the corresponding scatter plots. These results could suggest that the features were calculated from too short signals to preserve the mathematical relations. However, of note is the fact that the strong correlations between sTE with $E_{3}$ and $S_{T}$ with $E_{3}$ were still present in the graph representing relations estimated from the original signals. Hence, we had evidence that the relations between sTE, $S_{T}$, and $E_{3}$ could represent important physiological information.

**Figure 6:**
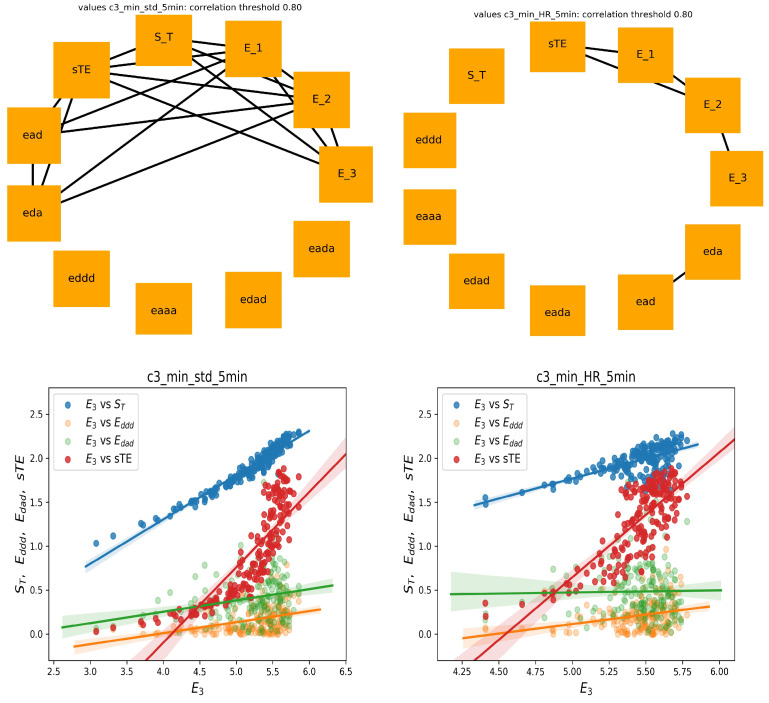
The graphs of strong correlations between entropic measures when five minute segments with the minimal stdRR (**left**) and minimal HR (**right**) are chosen for the analysis. Below, the scatter plots (with regression lines) between $E_{3}$ and other important entropic indices are shown.

Finally, let us show the correlation analysis performed for the features calculated from the five minute segment, which displayed a minimal stdRR for a given person; see Figure 6 (left). This graph shows many relations that were presented in the graph of 240 min series, including relations between sTE, $S_{T}$, $E_{ddd}$, and $E_{dad}$ with $E_{3}$. The significant reduction in total HRV, which corresponds to the segment with minimal stdRR, could correspond to the moments of transitions from NREM to REM sleep, where a shift of sympatho-vagal balance toward a vagal withdrawal and a possible sympathetic predominance is reported [12,34].

On the other hand, the graph corresponding to the five minute segments with the minimal meanHR (see Figure 6 (right)), together with the corresponding scatter plots, showed similarity to the graph constructed on base of the shuffled signal rather. Here, we did not observe the strong relationships between sTE and $S_{T}$ with $E_{3}$. This observation agrees with the common belief that during deep sleep, where the minimal HR was expected, the system was driven solely by the strong activity of the vagal nervous systems and that the sympathetic activity was switched off [12,13,34].

Concluding our observations on correlations among $E_{3}$ and sTE, $S_{T}$, $E_{ddd}$, and $E_{dad}$, we can hypothesize that the structure of these relationships can be an indicator of the sympathetic system activity.

### 3.4. Classification with SVM

Let us start with the presentation of the age dependence of each studied variable found by the regression analysis. In Table 2, we subsequently show the results of the normality test, the equal variance test, the age groups found significantly statistically different, and the linear regression results with the quality evaluated by the $R^{2}$ Pearson correlation coefficient and the two linear model coefficients with their statistical significance.

**Figure 7:**
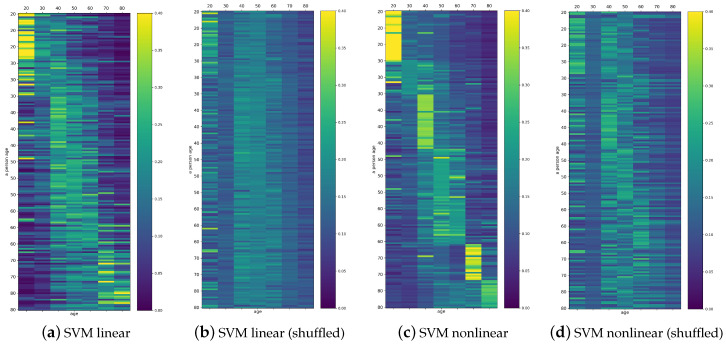
Typical matrix plots of probabilities provided by the decision functions of the applied classifiers. The results, obtained for 181 persons, are arranged according to the age decade of a person (vertical axis) and the age decade class (horizontal axis).

One can see from Table 2 that almost all studied features displayed a dependence on age. Therefore, one can expect that these features could serve as a good proposition for the automatic classification by SVM. We performed the classification with the linear SVM and with SVM acting on Gaussian kernels with γ=0.2 and regulation C=1. Each classifier constructed a decision function, which provided the probability that a given person belonged to the given age decade. Because of the stochastic methods used in probability estimates, each classifier run could provide a different result. Typical probabilities obtained for linear SVM and for nonlinear SVM when features were found from 240 min segments are listed graphically in Figure 7. Together, we show the validation of the obtained results by presenting probabilities found by the same classifiers for the shuffled signals.

One can learn from Figure 7 that, in general, the maximum for class belonging revealed the true person age decade in the case of many persons. Especially, the age seemed to be properly estimated in the groups of the signals describing young and elderly people. However, the classification of the adult persons, at the age group of 40’s, 50’s, or 60’s, was not clear. The maximal probability among these classes was not obvious. Consequently, the winning class could be incidental. Notice that this effect was evident in the case of the shuffled signals, independent of which classifier, linear or nonlinear, was applied.

**Figure 8:**
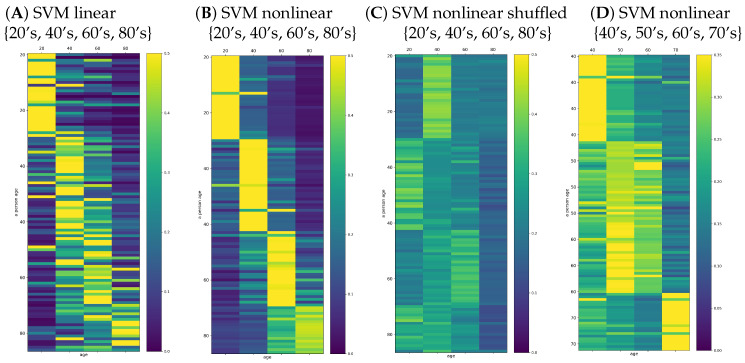
Typical matrix plots of the probabilities provided by the decision functions of the applied classifiers. Results for 107 of 181 persons (**A**–**C**) and for 134 of 181 persons (**D**) are arranged according to their age decade.

The improvement of the classifier quality could be observed when the classification task was limited to the four classes: 20’s, 40’s, 60’s, and 80’s. These results are shown in Figure 8A,B. The effort of the classification SVM algorithms can be evaluated by reading the classification outcomes provided by the shuffled signals; see Figure 8C. Additionally, we tested the improvement of classifiers when the classification task was restricted to the adult people: 40’s, 50’s, 60’s, and 70’s; Figure 8D. We see the essential refinement of the automatic classification: the wining class was evident in the case of the classes of 20’s, 40’s, 60’s, and 80’s. In particular, an average of 77.7±1.5 people (score =72.6±1.4%) were classified correctly by the linear SVM. In the case of nonlinear SVM classification, the winning class agreed almost everywhere with the true age decade of a person; on average, only seven incorrect classifications (score =93.6±5.3%). However, when the classification task was performed on the features of 40’s, 50’s, 60’s, and 70’s, the mean score =65.9±17.0% was lower and varied significantly from run to run, suggesting instability in the numerical estimates.

**Figure 9:**
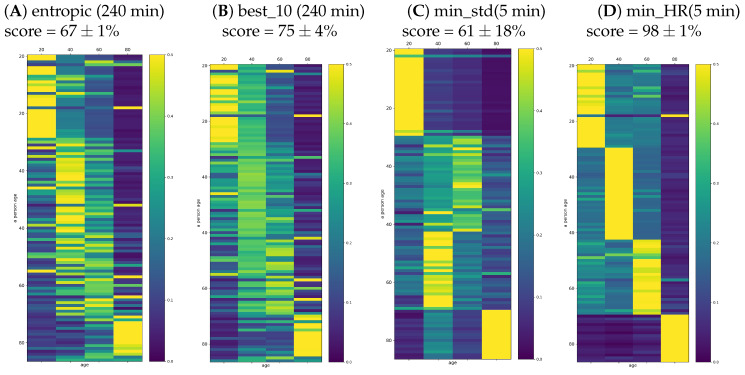
Typical matrix plots of decision function probability for SVM with the Gaussian kernel and γ=0.2. Here, classification results are given for different sets of features used in the classification: (**A**) entropic measures and (**B**) best_10 measures; and when five minute segments with the special characteristic were extracted: (**C**) minimal stdRR and (**D**) minimal HR.

One can worry that the automatic classification task based on 33 features in the population of 107 signals could not be properly fitted because the age groups were too small to couple with such a wide set of features. Therefore, in the plots of Figure 9, we report the results found when the set of features was restricted to (A) entropic indices (dynamical landscape and partial entropy) and (B) best_10 measures. The set of best_10 indices was constructed with the highest classification score achieved on the set of all signals. This set consisted of {meanRR, total, sd2, PAS, PSS, PIP, $E_{dad},E_{da},p\left(ad\right),p\left(ada\right)\}$. One can see that restriction of the set of features limited the classification quality, namely the score was significantly smaller than in the case when all features were taken into account.

In Figure 9C,D, we also show the probabilities provided by signals representing the five minute segments with minimal stdRR and with minimal HR. While the segments corresponding to the minimal HR provided a very accurate solution for the classification task, the segments extracted according to minimal stdRR correctly discerned only the young and elderly people.

## 4. Discussion and Conclusions

The intensive studies on healthy populations proved the dependence between the biological age of a human and many HRV indices [21,39,40,41,42,43,44]. A vivid discussion is running whether by this dependence, the assessment of the autonomic function can be achieved [17,18,45]. Consistent results have been obtained after autonomic provocations by chemical blockade of vagal or sympathetic activity [46] and due to postural change, which boosts the sympathetic tone [47,48]. However, it has been also suggested that HRV may be dominated by tissue properties rather than by ANS regulation [17,49]. Namely, the excitability of the sinoatrial node cell membrane could be claimed as a main source of HRV as it determines the organism’s homeostasis. In people who have had a heart transplant, one can observe the heart rhythm, which is shaped without the direct ANS control because of the denervation of the donor heart by surgical dissection of postganglionic neurons [50]. These rhythms occur different from the rhythms observed in the healthy people of a similar age, independently of how long after the surgery [29].

The external stressors such as structural heart disease, hypertension, and possibly diabetes are known to induce a slow, but progressive process of structural remodeling in the cardiac tissue [51,52]. Therefore, the abnormal levels of short term HRV indices observed are supposed to be related to so-called erratic rhythms, i.e., rhythms probably resulting from remodeling of the cardiac tissue [10,21,53,54,55]. Accordingly, the higher HRV values cannot be attributed solely to the better organization of the feedback reflexes driving the organism’s response to the actual body needs, but rather, the characteristics of the cardiac cells and the structure of their interconnections should be taken into account [56].

In the following, the specially chosen ML methods were applied to the set of thirty three features, HRV indices, estimated from 240 min nocturnal recordings of 181 healthy people of different ages to test whether the automated methods of ML could advance the research on separating HRV indices into those of ANS origin and the erratic part. The choice of sleeping period for the analysis was motivated by the limitation of possible artifacts. However, also, the nocturnal rest displayed a special organization in which the time periods with strong vagal activity and strong withdrawal of sympathetic activity of deep sleep were switched into REM periods where ANS activity was similar to the awake state [13,34]. Although a discussion on this subject is beyond the scope of this article, it is worth noting that our analysis could directly benefit from this specific nocturnal ANS activity; we have found arguments supporting the basic concepts of HRV:

- the five factors identified by FA could have physiological meaning, the three of them relying on the pattern indices;
- the period corresponding to the lowest HR might be associated with deep sleep where autonomic regulation is restricted to the vagal activity;
- the strong correlation between sTE and $S_{T}$ with $E_{3}$ can be hypothesized as the fingerprint of the sympathetic activity.

In particular, we found that entropic indices operating on the whole set of patterns: the dynamic landscape measures, refer to the vagal activity rather, while the corresponding counting measures describe the sympathetic-vagal balance in ANS. Therefore, both characterizations: the total volume of patterns, as well as their distribution are important in studies of ANS activity as they describe distinct aspects of the ANS control organization.

We have found the ML methods to be advancing versatile validations of the known results and common intuitions. We were allowed to practice comprehensively, to verify many aspects of the studied phenomena in an unlimited way. In particular, we utilized the flexibility of the ML methods, using the shuffling as a validation method. However, also, we were concerned about them to avoid possible pitfalls. Additionally, what is extremely profitable, we were given attractive frames for the presentation of the results.

ML analysis issued a warning about the use of short segments of recordings in research based on statistical properties. Many of the HRV measures rely on features constructed following the assumption that the signals are stationary. However, RR intervals are not stationary, which was proven by many methods. Accordingly, the HRV measures estimated from short signals may overestimate the role of fluctuations, and the results are overtaken by incidental events. We observed this effect while testing measures revealing pattern statistics. We found that the presence or absence of a strong correlation between some pattern HRV indices could indicate a specific dynamical order, which was attributed to the original heart rhythms only. However this arrangement was seen only with sufficiently long signals. However, under the controlled conditions, such as minimal meanHR or maximal stdRR, the short signals provided a satisfactory description of the corresponding physiological state.

The collection of features obtained for one person from the subsequent five minute segments might be the source data for other types of analysis: the stroboscopic approach, which could assign a new role to short segments. The deep learning methods can be applied, and different insights into the organization of the dynamics in RR intervals can be offered.

Supposing that the dynamic organization of RR intervals was age dependent, the classification with SVM was performed. However, the methods of classifications used by us seemed to fail with our data. Although most of studied indices displayed dependence on age, the decision functions of the SVM methods applied to these indices were proven weak in their ability to discern the age. The methods, in general, recognized the group’s decade, but belonging to the group was not obvious. Probably, the set of considered signals was too small.

## Figures and Tables

**Table 1:** The factor design as the coefficients of linear combinations of the investigated features found in 240 min signals. The coefficients in parentheses are obtained for signals with shuffled values. For each index, its maximal value is bold. Below the factor name, the percent of the explained variance by this factor is given.

Index Name	Factor I	Factor II	Factor III	Factor IV	Factor V
% Variance	40 (36)	22 (27)	17 (12)	7 (12)	6 (5)
meanRR	0.37 (0.11)	0.28 (0.32)	0.02 (0.13)	**0.77 (0.93)**	0.18 (0.02)
meanHR	−0.36 (−0.10)	−0.25 (−0.23)	−0.01 (−0.11)	**−0.78 (−0.95)**	−0.11 (−0.01)
stdRR	0.50 (0.36)	0.22 **(0.87)**	0.27 (0.21)	0.21 (0.25)	**0.76** (0.08)
sd2	0.46 (0.36)	0.22 **(0.87)**	0.29 (0.21)	0.21 (0.25)	**0.77** (0.08)
total	**0.69** (0.08)	−0.09 (0.31)	0.20 (0.14)	0.06 **(0.93)**	−0.43 (0.01)
rVLF	**−0.70** (0.10)	0.01 (0.04)	−0.27 (0.09)	0.30 (0.10)	0.37 (0.09)
rLF	−0.07 (0.00)	−0.12 (0.08)	**0.75** (0.01)	−0.09 (−0.02)	−0.06 **(0.84)**
rHF	**0.70** (−0.06)	0.07 (−0.05)	−0.37 (−0.07)	−0.19 (−0.05)	−0.27 **(−1.00)**
pNN50	**0.91 (0.67)**	0.15 (0.58)	0.01 (0.38)	0.09 (0.22)	0.23 (0.03)
pNN20	**0.92 (0.71)**	0.16 (0.50)	0.09 (0.42)	0.29 (0.23)	0.06 (0.30)
RMSSD	**0.93** (0.37)	0.18 **(0.87)**	0.06 (0.20)	0.03 (0.25)	0.25 (0.07)
sd1	**0.93** (0.37)	0.18 **(0.87)**	0.06 (0.20)	0.03 (0.25)	0.25 (0.07)
$E_{3}$	**0.91 (0.74)**	0.16 (0.17)	0.21 (0.43)	0.25 (−0.36)	0.05 (0.01)
$E_{2}$	**0.94** (0.59)	0.16 (**0.69)**	0.14 (0.34)	0.20 (0.20)	0.11 (0.03)
$E_{1}$	**0.95** (0.51)	0.18 (**0.76)**	0.13 (0.30)	0.19 (0.25)	0.12 (0.05)
$S_{T}$	**0.94 (0.70)**	0.13 (0.55)	0.16 (0.40)	0.22 (0.10)	0.11 (0.01)
sTE	**0.92** (0.53)	0.11 **(0.72)**	−0.03 (0.30)	0.07 (0.29)	0.28 (0.03)
n_zero	**−0.85 (−0.73)**	−0.17 (−0.45)	−0.15 (−0.44)	−0.35 (−0.24)	0.03 (−0.03)
PSS	−0.10 (−0.43)	0.35 (−0.48)	**−0.93 (−0.72)**	−0.03 (−0.25)	−0.07 (−0.07)
p(ddd)	0.02 (0.44)	−0.46 (0.41)	**0.77 (0.63)**	0.10 (0.22)	−0.04 (0.07)
$E_{ddd}$	0.13 (0.50)	−0.43 (0.41)	**0.80 (0.65)**	0.14 (0.13)	0.01 (0.07)
$E_{aaa}$	0.29 (0.46)	−0.17 (0.49)	**0.86 (0.59)**	0.00 (0.17)	0.21 (0.05)
p(aaa)	0.16 (0.39)	−0.21 (0.50)	**0.90 (0.57)**	−0.04 (0.25)	0.16 (0.05)
PAS	0.07 **(0.91)**	**0.95** (0.32)	−0.26 (0.14)	0.08 (0.16)	0.08 (0.06)
$E_{dad}$	0.34 **(0.91)**	**0.85** (0.27)	−0.22 (0.16)	0.18 (−0.03)	0.08 (0.06)
p(dad)	0.13 **(0.88)**	**0.89** (0.29)	−0.30 (0.26)	0.15 (0.17)	0.08 (0.08)
p(ada)	0.02 **(0.86)**	**0.93** (0.35)	−0.23 (0.22)	0.02 (0.14)	0.08 (0.03)
$E_{ada}$	0.24 **(0.89)**	**0.91** (0.31)	−0.17 (0.31)	0.07 (−0.06)	0.08 (0.02)
PIP	0.53 **(0.87)**	**0.69** (0.39)	−0.39 (0.22)	0.25 (0.20)	0.02 (0.03)
$E_{ad}$	**0.81 (0.64)**	0.46 **(0.66)**	−0.23 (0.31)	0.26 (0.21)	0.08 (0.03)
p(ad)	0.51 **(0.82)**	**0.65** (0.40)	−0.43 (0.22)	0.30 (0.24)	0.00 (0.02)
p(da)	0.53 **(0.86)**	**0.73** (0.36)	−0.34 (0.22)	0.18 (0.15)	0.03 (0.03)
$E_{da}$	**0.81 (0.66)**	0.53 **(0.65)**	−0.14 (0.32)	0.14 (0.19)	0.10 (0.03)

**Table 2:** The linear regression analysis of the discussed features.

Index Name	W-S Test	B-F Test	ANOVA Test	Significantly Different Groups	$R^{2}$	$a_{0}^{P}$	$a_{1}^{P}$
meanRR	+	+	−	no groups	0.023 ${}^{*}$	987 ${}^{\#}$	−0.850 ${}^{*}$
meanHR	−	+	−	no groups	$0.017^{NS}$	62.4 ${}^{\#}$	0.050 ${}^{NS}$
stdRR	−	−	+	20vs.80,70,6030vs.70	0.138 ${}^{\#}$	115 ${}^{\#}$	−0.651 ${}^{\#}$
sd2	−	−	+	20vs.80,70,6070vs.30,40	$0.131^{\#}$	158 ${}^{\#}$	−0.878 ${}^{\#}$
total	+	+	+	20vs.80,…,4080vs.30,40	$0.190^{\#}$	35.9 ${}^{\#}$	−0.135 ${}^{\#}$
rVLF	+	+	+	20vs.50	0.057 ${}^{*}$	0.301 ${}^{\#}$	0.001 ${}^{*}$
rLF	−	−	+	no groups	0.052 ${}^{*}$	0.388 ${}^{\#}$	−0.001 ${}^{*}$
rHF	+	+	+	50vs.20,70	0.002 ${}^{NS}$	0.311 ${}^{\#}$	−0.0001 ${}^{NS}$
pNN50	−	−	+	20vs.80,…,50	0.211 ${}^{\#}$	$29.9^{\#}$	−0.346 ${}^{\#}$
pNN20	+	+	+	20vs.80,…,4080vs.30,40	0.224	68.1 ${}^{\#}$	−0.49 ${}^{\#}$
RMSSD	−	−	+	20vs.80,…,40	0.173 ${}^{\#}$	56.1 ${}^{\#}$	−0.407 ${}^{\#}$
sd1	−	−	+	20vs.80,…,40	0.173 ${}^{\#}$	39.6 ${}^{\#}$	−0.288 ${}^{\#}$
$E_{3}$	−	+	−	20vs.80,…,5030vs.80	0.213 ${}^{\#}$	8.71 ${}^{\#}$	−0.026 ${}^{*}$
$E_{2}$	+	+	+	20vs.80,…,4030vs.80	0.221 ${}^{\#}$	6.473 ${}^{\#}$	−0.024 ${}^{\#}$
$E_{1}$	+	+	+	20vs.80,…,4030vs.80	0.205 ${}^{\#}$	3.302 ${}^{\#}$	−0.012 ${}^{\#}$
$S_{T}$	+	+	+	20vs.80,…,4030vs.80	0.236 ${}^{\#}$	3.171 ${}^{\#}$	−0.012 ${}^{\#}$
sTE	−	−	−	20vs.80,…,4030vs.80	0.218 ${}^{\#}$	0.935 ${}^{\#}$	−0.009 ${}^{\#}$
n_zero	−	+	+	20vs.80,…,4030vs.80	0.186 ${}^{\#}$	0.065 ${}^{\#}$	0.002 ${}^{\#}$
PSS	−	+	−	80vs.30,40,5070vs.30,40,50	0.068 ${}^{\#}$	0.876 ${}^{\#}$	0.0007 ${}^{\#}$
p(ddd)	−	+	+	80vs.50,40,3070vs.50,40	0.066 ${}^{\#}$	0.066 ${}^{\#}$	−0.0003 ${}^{\#}$
$E_{ddd}$	−	+	+	80vs.50,…,2070vs.50,40,30	0.104 ${}^{\#}$	0.565 ${}^{\#}$	−0.003 ${}^{\#}$
$E_{aaa}$	−	+	+	80vs.50,…,20	0.085 ${}^{\#}$	0.578 ${}^{\#}$	−0.003 ${}^{\#}$
p(aaa)	−	+	+	50vs.80	0.053 ${}^{*}$	0.067 ${}^{\#}$	−0.0003 ${}^{*}$
PAS	−	+	+	80vs.50,40,3070vs.50	0.064 ${}^{\#}$	0.103 ${}^{\#}$	0.0009 ${}^{\#}$
$E_{dad}$	−	+	+	80vs.50	$0.004^{NS}$	0.546 ${}^{\#}$	0.001 ${}^{NS}$
p(dad)	−	+	+	80vs.50,40,3070vs.50	0.040 ${}^{*}$	0.057 ${}^{\#}$	$0.0004^{*}$
p(ada)	−	+	+	80vs.50,…,20,70vs.50,30	0.084 ${}^{\#}$	0.046 ${}^{\#}$	0.0006 ${}^{\#}$
$E_{ada}$	−	+	+	80vs.50,40,30	0.025 ${}^{*}$	0.455 ${}^{\#}$	0.0026 ${}^{*}$
PIP	+	+	+	20vs.50	0.002 ${}^{NS}$	0.416 ${}^{\#}$	−0.0002 ${}^{NS}$
$E_{ad}$	−	+	+	20vs.40,…,80	0.085 ${}^{\#}$	1.446 ${}^{\#}$	−0.006 ${}^{\#}$
p(ad)	+	+	+	20vs.50	0.006 ${}^{NS}$	0.215 ${}^{\#}$	−0.0001 ${}^{NS}$
p(da)	+	+	+	50vs.20,80	0.0004 ${}^{NS}$	0.201 ${}^{\#}$	−0.00004 ${}^{NS}$
$E_{da}$	−	+	+	20vs.50,60	0.060 ${}^{\#}$	1.357 ${}^{\#}$	−0.005 ${}^{\#}$

Notation used for the quantification of statistical significance: #: p<0.001, *: p<0.05, NS: p≥0.05. W-S test: result of the Wilk–Shapiro test for normality: + passed, − failed; B-F test: result of the Brown–Forsythe test for equal variance: + passed, − failed; age groups found significantly different by ANOVA or Kruskal–Wallis ANOVA on ranks in case W-S failed; $R^{2}$ Pearson correlation coefficient for the estimated linear regression with its statistical significance; $a_{0}^{P}$ the intercept value with its statistical significance; $a_{1}^{P}$ the linear regression coefficient with its statistical significance.

## Abbreviations

The following abbreviations are used in this manuscript:

HRV	Heart rate variability
ANS	Autonomic nervous system
ML	Machine learning
FA	Factor analysis
SVM	Support vector machine
